# Determinants of aortic growth rate in patients with bicuspid aortic valve by cardiovascular magnetic resonance

**DOI:** 10.1136/openhrt-2019-001095

**Published:** 2019-11-02

**Authors:** Froso Sophocleous, Bostjan Berlot, Maria Victoria Ordonez, Mai Baquedano, Elena Giulia Milano, Viola De Francesco, Graham Stuart, Massimo Caputo, Chiara Bucciarelli-Ducci, Giovanni Biglino

**Affiliations:** 1 Bristol Medical School, University of Bristol, Bristol, UK; 2 Bristol Heart Institute, University Hospitals Bristol NHS Foundation Trust, Bristol, UK; 3 Institute of Cardiovascular Science, University College London, London, UK; 4 Department of Surgery, Dentistry, Paediatrics and Gynaecology, University of Verona, Verona, Italy; 5 National Heart and Lung Institute, Imperial College London, London, UK

**Keywords:** bicuspid aortic valve, aortic growth, aortic root, proximal ascending aorta, coarctation, valve morphotype

## Abstract

**Objectives:**

This study aimed to identify determinants of aortic growth rate in bicuspid aortic valve (BAV) patients. We hypothesised that (1) BAV patients with repaired coarctation (CoA) exhibit decreased aortic growth rate, (2) moderate/severe re-coarctation (reCoA) results in increased growth rate, (3) patients with right non-coronary (RN) valve cusps fusion pattern exhibit increased aortic growth rate compared with right-left cusps fusion and type 0 valves.

**Methods:**

Starting from n=521 BAV patients with cardiovascular magnetic resonance data, we identified n=145 patients with at least two scans for aortic growth analysis. Indexed areas of the sinuses of Valsalva and ascending aorta (AAo) were calculated from cine images in end-systole and end-diastole. Patients were classified based on dilation phenotype, presence of CoA, aortic valve function and BAV morphotype. Comparisons between groups were performed. Linear regression was carried out to identify associations between risk factors and aortic growth rate.

**Results:**

Patients (39±16 years of age, 68% male) had scans 3.7±1.8 years apart; 32 presented with AAo dilation, 18 with aortic root dilation and 32 were overall dilated. Patients with repaired CoA (n=61) showed decreased aortic root growth rate compared with patients without CoA (p≤0.03) regardless of sex or age. ReCoA, aortic stenosis, regurgitation and history of hypertension were not associated with growth rate. RN fusion pattern showed the highest aortic root growth rate and type 0 the smallest (0.30 vs 0.08 cm^2^/m*year, end-systole, p=0.03).

**Conclusions:**

Presence of CoA and cusp fusion morphotype were associated with changes in rate of root dilation in our BAV population.

**Key questions**

**What is already known about this subject?**

- Patients with bicuspid aortic valve (BAV) present with different degrees of aortic dilation. Despite known phenotypic variations in BAV aortopathy, and differences in aortic architecture in BAV patients with concomitant aortic coarctation (CoA), determinants of aortic growth rate are not fully known.

**What does this study add?**

- This study highlights differences in BAV patients with/without CoA based on cardiovascular magnetic resonance imaging data, identifying particularly differences in root dilation. The study also reinforces previous echocardiographic observations on the association between BAV morphotype and differences in aortic dilation, using a different imaging modality.

**How might this impact on clinical practice?**

- This study suggests that CoA patients with concomitant BAV disease could be treated as a separate group with a different phenotype, thus refining risk stratification and monitoring of potential progression of BAV aortopathy.

## Introduction

Bicuspid aortic valve (BAV) is a highly heterogeneous congenital heart disease (CHD), commonly associated with increased risk of developing thoracic aortic aneurysms and acute aortic dissection,^1^ the latter increasing after aortic valve replacement (AVR).^2^ BAV aortopathy can range from slow-asymptomatic aortic diameter growth to rapid progression or early-life threatening aortic complications.^3^ In vitro and in vivo studies have explored the inherent defect of aortas in BAV patients leading to altered wall mechanical properties which contribute to aneurysm formation.^4 5^ However, at present there is no established risk marker to help in the prognosis of BAV aortic growth.^6^ Known predictors of BAV aortopathy progression include older age, male sex, increased systolic blood pressure, valvular dysfunction and BAV morphology.^7 8^ While no consistent correlation has been reported between BAV morphotype and aortopathy pattern,^9^ the phenotype of dilation is reported to be predictive of the disease course, and thus being used to facilitate risk stratification and standardise surgical approaches.^10 11^

BAV is often concomitant with aortic coarctation (CoA)^12^ and aortic wall complications are frequent in adults with CoA,^13^ whereas those with progressive course of the disease may also develop aortic dilation and be at risk of aneurysm and aortic dissection. Recent studies have shown that ascending and post-CoA aortic diameters or dilations are linked to the degree of CoA severity^14^ and that, in a CoA population, patients with BAV were more likely to have moderate or severe ascending aorta (AAo) dilation compared with those with a trileaflet valve.^15^ While BAV-aortopathy is likely a reflection of BAV morphotype rather than CoA or its physiological effects,^16^ questions remain about changes in aortic growth in BAV+CoA patients compared with isolated BAV. Furthermore, the coexistence of CoA with BAV has been associated with increased risk of aortic dissection compared with patients with isolated CoA,^17^ but does aortic growth rate vary compared with patients with isolated BAV?

Taking advantage of a large cohort of BAV patients with and without CoA, this study aims to assess the effect of CoA and other potential determinants on aortic growth rate. Based on a recent study from our group reporting morphological differences in the aortic arch architecture in BAV patients with/without CoA,^18^ the primary hypothesis is that BAV+CoA patients exhibit slower aortic growth rate, while patients with re-coarctation (reCoA) exhibit increased aortic growth rate. Furthermore, based on differences in aortic growth across BAV fusion types established with echocardiography,^19^ a secondary hypothesis is that patients with right non-coronary (RN) valve fusion type exhibit increased aortic growth rate.

## Methods

### Patient population

**Figure 1:**
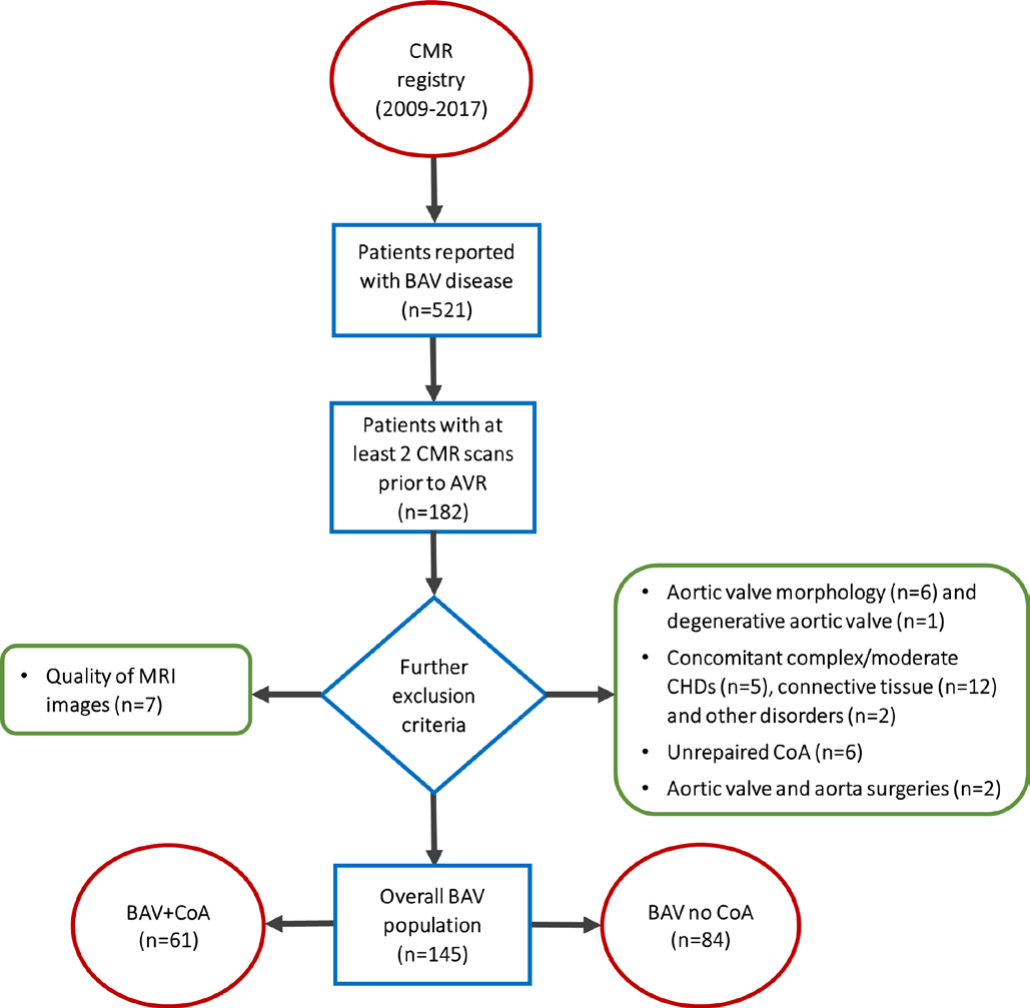
Patient selection. Inclusion criteria: two CMR scans prior to AVR (n = 182). Exclusion criteria: suboptimal quality of CMR cine images (n=7), unconfirmed bicuspid morphology (n=6), degenerative aortic valve, any concomitant either complex or moderate CHDs, including Shone’s complex (n=2), tetralogy of Fallot (n=2) and Epstein’s anomaly (n=1); connective tissue disorders, including Marfan (n=1), Turner (n=10) and Ehlers Danlos (n=1) syndromes; pseudo-CoA (n=1), Kawasaki disease (n=1), unrepaired CoA (n=6) and surgeries (n=2) such as aortic valvotomy and aortic arch reconstruction. AVR, aortic valve replacement; BAV, bicuspid aortic valve; CMR, cardiovascular magnetic resonance; CHD, congenital heart disease.

This was a longitudinal single-centre retrospective study. Consecutive BAV patients (n = 521) were identified in the cardiovascular magnetic resonance (CMR) imaging database between 2009 and 2017, of which 182 patients had CMR scans at two or more time-points before AVR. When patients had more than two scans available, the least and the most recent were considered. Exclusion criteria included: suboptimal image quality (n=7), unconfirmed bicuspid morphology (n=6, even after consulting trans-thoracic/trans-oesophageal echocardiography or CT angiography when in doubt), degenerative aortic valve (n=1), concomitant either moderate or complex CHD (n=2 Shone’s complex, n=2 Tetralogy of Fallot and n=1 Epstein’s anomaly), connective tissue disorders (n=1 Marfan, n=10 Turner and n=1 Ehlers Danlos syndromes, confirmed by genetic results), other diseases (n=1 pseudo-CoA, n=1 Kawasaki disease), unrepaired CoA (n=6) and surgeries structurally affecting the aortic valve and/or the aorta (n=1 aortic valvotomy and n=1 aortic arch reconstruction). A flowchart is provided in figure 1. All CMR data were acquired at 1.5 T (Avanto, Siemens Healthineers, Erlangen, Germany). Demographic and functional variables were collected from CMR reports, including age, height, sex, aortic valve morphology, severity of aortic regurgitation or stenosis, presence of repaired CoA, degree of reCoA and history of hypertension. The population was then divided into two subgroups, that is, patients with isolated BAV and patients with BAV and repaired CoA.

### Aortic measurements

**Figure 2:**
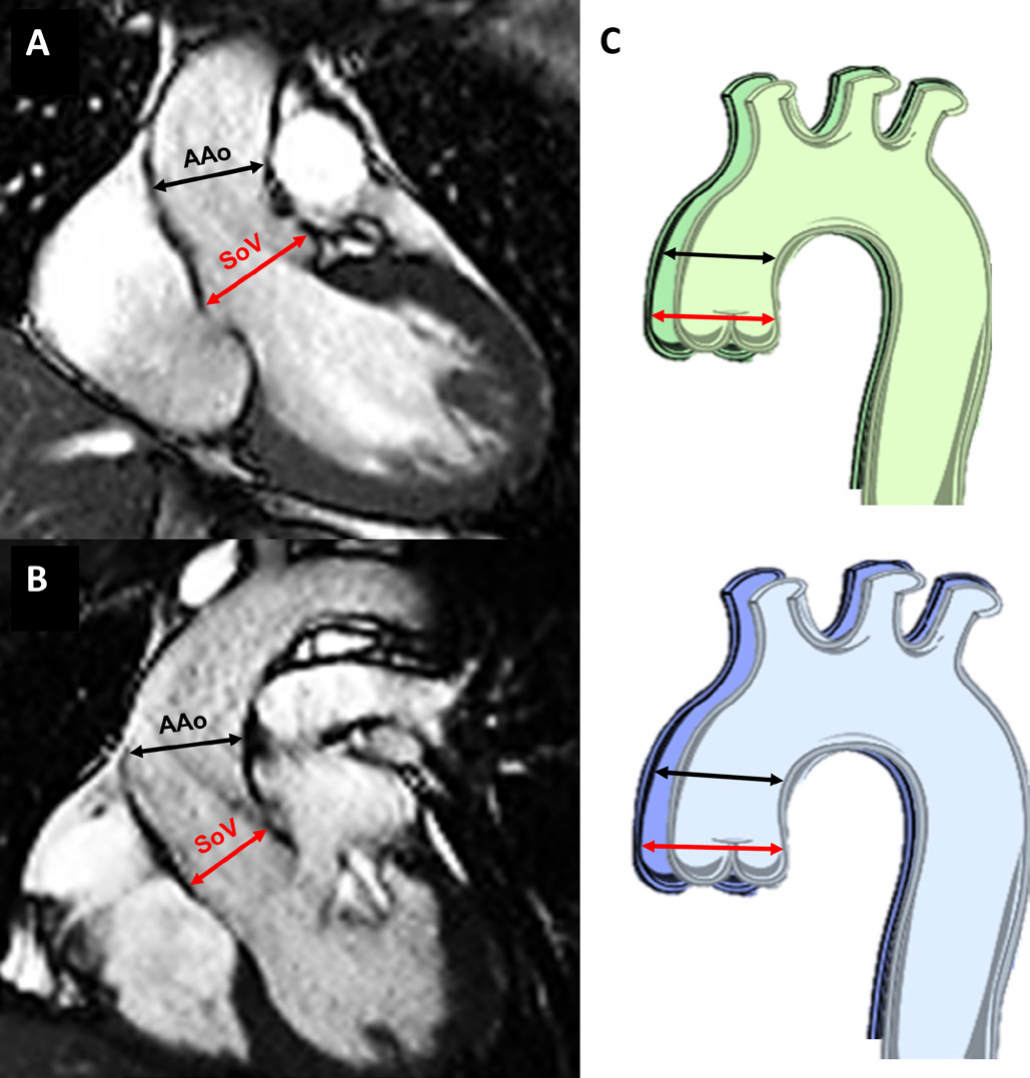
CMR measurements. Cine images showing measurements of aortic diameters, (A) at 3C and (B) at left ventricular outflow tract view. The red lines show the diameters measured at the SoV level and the black lines at the proximal AAo. (C) Schematic representation of changes at end-diastole (green) and end-systole (blue) over time to capture the possible dynamic nature of dimensional changes. AAo, ascending aorta; SoV, sinuses of Valsalva.

The aortic measurements were performed in the steady-state free precession cine images acquired in the three-chamber (3C) and left ventricular outflow tract (LVOT) views and in the oblique sagittal views, both at the level of the sinuses of Valsalva (SoV) and at the proximal AAo (figure 2). The axial views of the AAo were acquired using multiple planes resulting in true perpendicular planes, to consider possible dynamic changes over time and possible asymmetry of the aorta in keeping with the 2017 European Society of Cardiology Guidelines on Valvular Heart Disease.^20^ The AAo measurement was taken at the level of the right pulmonary artery. Having verified intra-observer and inter-observer reproducibility on 20 random cases (intraclass correlation coefficient=0.988 and 0.967, respectively), all measurements were taken by a single operator, who received the anonymised cases in random sequence and was blinded as to the history of CoA. Aortic growth rate was defined as the difference in indexed aortic area over the two time-points, having normalised aortic area by height^21^:

$$Growth\;Rate=\frac{\frac{A2}{H}-\frac{A1}{H}}{t2-t1}$$

where A, area; H, height; t, time. In order to avoid the assumption of circularity, the aortic radius was derived as the average of the diameters at 3C and LVOT and thus area calculated as:

$$A=\pi (\frac{D\left(3C\right)}{2}*\frac{D\left(LVOT\right)}{2})$$

where D, diameter.

### Variables and classification

**Figure 3:**
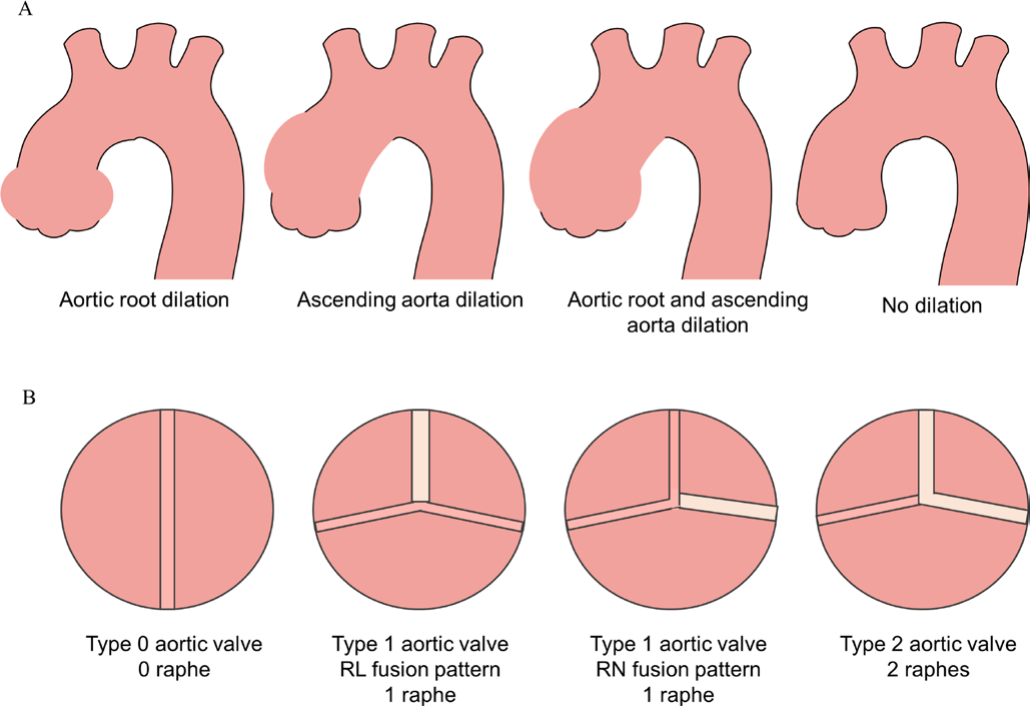
Aortic dilation phenotypes and bicuspid aortic valve (BAV) morphotypes. (A) Classification of the aortic dilation configurations in our population showing aortic root dilation, ascending aorta dilation, aortic root and ascending aorta dilation, and no dilation. (B) Classification of the BAV morphotypes showing type 0 valve with no raphe, type 1 valve with one central raphe having in one case a right coronary and left coronary fusion pattern and in the other case a right non-coronary fusion pattern, and type 2 valve with two raphes.

Valve morphotype was classified as ‘type 0’ (ie, true BAV with no raphe), ‘type 1’ (ie, one central raphe) and ‘type 2’ (ie, two central raphes). Furthermore, type 1 valves were subdivided intro right coronary and left coronary (RL) fusion patter, right coronary and RN fusion pattern and left coronary and left non-coronary (LN) fusion pattern.^9^ ReCoA was defined as a recurrence of narrowing at the CoA site. The severity of reCoA was assessed from the ratio of the isthmus diameter to the descending thoracic aorta, and classified as severe if <0.55, moderate to mild if 0.55–0.85 and absent if >0.85. The severity of aortic stenosis and aortic regurgitation were extracted from CMR reports and, for the purposes of this analysis, patients were classified as either having none-to-mild or moderate-to-severe stenosis/regurgitation. In addition, according to a modified Fazel classification of aortic dilation configurations^10 22^ patients were classified as presenting (1) increased SoV diameter, (2) increased AAo diameter, (3) increased SoV and AAo diameters, and (4) no substantial aortic growth, as summarised in figure 3. We classified patients as ‘no growth’ when aortic growth rate was <0.08 cm^2^/m*year, corresponding to a diameter change of <1.5 mm (ie, pixel spacing).

### Statistical analysis

Continuous variables are presented as mean±SD and categorical variables as counts or proportions. Normality was assessed with Shapiro test. Continuous variables were compared with a Mann-Whitney test, or with Kruskal-Wallis test and Dunn’s test for post hoc adjustment when comparing more than two groups (eg, valve morphotypes). Categorical variables were compared with χ^2^ test. The association between demographic/clinical variables and aortic growth rate was assessed using linear regression, and only those variables showing a significant association on a univariate model were further tested in a multivariate regression model. A p value <0.05 was considered an indicator of statistical significance, except for multiple comparisons where Bonferroni correction was applied. The analysis was carried out in R (R Foundation for Statistical Computing, Vienna, Austria).

## Results

A population n=145 BAV patients with two CMR scans was identified (age 39±16 years, 68% male). For each patient, the time interval between CMR scans was 3.7±1.8 years (range 0.3–7.7). Also, at baseline BAV+CoA patients were significantly younger age (34±13 vs 42±17, p=0.006). Patients’ characteristics are reported in table 1.

**Table 1:** Patient characteristics

Demographic and clinical variables	Repaired CoA, n=61	NoCoA, n=84	P value
Time gap between CMR scans	4.6±1.5 (range: 1.3–7.7)	3.0±1.9 (range: 0.3–7.7)	<0.001***
Mean age at baseline	34±13 (range: 13–60)	42±17 (range: 15–73)	0.006**
Mean age at last follow-up	38±13 (range: 18–64)	45±17 (range: 17–77)	0.02*
Males	n=36, 59%	n=62, 74%	0.06
ReCoA	n=33, 54%	–	N/A
Moderate/severe reCoA	n=12, 36%	–	N/A
Valve type 0	n=4, 7%	n=11, 13%	0.01*
Valve type 1, RL fusion	n=51, 84%	n=52, 62%	
Valve type 1, RN fusion	n=3, 5%	n=15, 18%	
Moderate/severe aortic stenosis	n=3, 5%	n=26, 31%	<0.001***
Moderate/severe aortic regurgitation	n=4, 7%	n=19, 22%	0.009**
Hypertensive patients	n=26, 43% (22 under medication, three no medication and one unknown)	n=32, 38% (30 are under medication and two unknowns)	0.5
Type of CoA repair	End-to-end (n=33) Subclavian flap (n=9) Patch (n=7) Stent (n=3) Interposition graft (n=2)		N/A

CMR, cardiovascular magnetic resonance; CoA, coarctation; reCoA, re-coarctation; RL, right coronary and left coronary; RN, right non-coronary.

The most common BAV morphotype was the type 1 (83%), predominantly with RL fusion type (71%). Overall, patients presented with varying degrees of aortic stenosis (none: n=87, mild: n=29, moderate: n=20, severe: n=9) and aortic regurgitation (none: n=72, mild: n=50, moderate: n=14, severe: n=9), and patients with isolated BAV presented more frequently with severe stenosis and/or regurgitation (table 1). There was no difference in history of hypertension between isolated BAV and BAV+CoA patients.

Overall, we classified 63 BAV patients without aortic dilation, 32 with AAo dilation, 18 with aortic root dilation and 32 with overall dilated aorta, table 2. In the CoA subgroup, 54% patients had reCoA, of which 36% were moderate-to-severe.

**Table 2:** Distribution of patients according to aortic dilation phenotype and BAV morphotype

End-systole		No dilation	AAo dilation	Root dilation	Overall dilated
CoA	Type 0	3	0	0	1
	Type 1, RL	30	11	4	6
	Type 1, RN	1	0	2	0
	Type 1, LN	2	1	0	0
	Type 2	0	0	0	0
No CoA	Type 0	5	4	1	1
	Type 1, RL	15	14	8	15
	Type 1, RN	4	2	3	6
	Type 1, LN	0	0	0	2
	Type 2	3	0	0	1
Total		**63**	**32**	**18**	**32**

CoA, coarctation; LN, left non-coronary; RL, right coronary and left coronary; RN, right non-coronary.

Aortic growth rate in SoV was significantly lower in BAV+CoA patients compared with isolated BAV (end-diastole: 0.05±0.09 vs 0.13±0.2 cm^2^/m*year, p=0.03; end-systole: 0.05±0.09 vs 0.19±0.3 cm^2^/m*year, p=0.005). Aortic growth rate in SoV at end-systole also varied significantly according to BAV morphotype, with RN fusion pattern showing the highest growth and type 0 the smallest (0.3±0.5 vs 0.1±0.2 vs 0.08±0.1 cm^2^/m*year, p=0.03). An in-between groups analysis showed a significant difference between type 1 RL and RN valves (p=0.006) and between type 0 valves and type 1 RN valves (p=0.01). No correlation observed between aortic growth rate, age and sex (table 3).

**Table 3:** Results of univariate linear regression analysis assessing the association of demographic/clinical variables and aortic growth rate

Demographic and clinical variables	End-systole		End-diastole	
	SoV	AAo	SoV	AAo
Age at baseline	p=0.7	p=0.7	p=0.9	p=0.6
Age at last follow-up	p=1	p=0.5	p=0.8	p=0.4
Sex	p=0.5	p=0.1	p=0.8	p=0.4
Presence of CoA	p=0.004**	p=0.1	p=0.01*	p=0.05
ReCoA	p=0.8	p=0.9	p=0.5	p=0.7
Severity of reCoA	p=0.9	p=0.8	p=0.5	p=0.4
Valve morphotype	p=0.008**	p=0.07	p=0.01*	p=0.07
Severity of aortic stenosis	p=0.8	p=0.6	p=0.8	p=1
Severity of aortic regurgitation	p=0.4	p=0.5	p=0.3	p=0.7
History of hypertension	p=0.6	p=0.3	p=0.5	p=0.4

AAo, ascending aorta; CoA, coarctation; reCoA, re-coarctation; SoV, sinus of Valsalva.

Results from regression analysis are reported in table 3. ReCoA, severity of aortic stenosis and regurgitation, and history of hypertension were not associated with aortic growth rate, neither in the overall population nor in the BAV+CoA subgroup. In patients with CoA, type of surgical repair and age of repair did not correlate with aortic growth rate (p≥0.16 and p≥0.33, respectively) over this time window.

Multiple regression analysis showed that the concomitant absence of CoA with type 1 valve with RN fusion pattern in end-systole results in a 0.3 cm^2^/m*year increase in aortic root growth rate. From a clinical standpoint, changes in aortic diameter guide the decision to intervene, and this corresponds to 1 mm/year increase in aortic diameter, compared with 0.5 mm/year for type 1 valve with RL fusion pattern and 0.3 mm/year for type 0 valve.

All diameters, area values and aortic growth rate as indexed area of AAo and SoV both in end-systole and end-diastole, corresponding to the subgroups of CoA, NoCoA, reCoA, vale type 0, type 1 with RL cusp fusion and RN cusp fusion are reported in the (online supplementary material).

10.1136/openhrt-2019-001095.supp1 Supplementary data

## Discussion

This longitudinal study uses CMR data to explore changes in aortic growth rate in a cohort of BAV patients. Results showed that patients with repaired CoA tended to have slower aortic root growth rate compared with patients with isolated BAV. Previous work from our group based on a statistical shape modelling framework^18^ revealed nuanced differences in arch morphology in BAV patients with/without CoA, suggesting detrimental functional implications for some aortic arch architectures, and with BAV+CoA patients generally presenting with smaller ascending and larger descending aortas compared with isolated BAV. This agrees with observations in the literature that prior CoA repair may protect BAV aorta from rapid dilation.^8^ With regards to functional implications, a recent echocardiographic study of 631 BAV patients with and without CoA found that BAV patients with CoA were associated with increased risk of AAo complications, smaller aortic root dimensions and less severe valvular dysfunction compared with isolated BAV patients over a similar time window to that of our study, but this study did not differentiate repaired and unrepaired CoA patients.^23^

Our observations on growth rate, translated into yearly increase in aortic diameter, indicate that an unfavourable combination of type 1 valve with RN fusion in patients without CoA can result in a substantial increment in diameter of 1 mm/year, compared with valve type 0 where the root diameter increase was found to be 0.3 mm/year. Although seemingly small, these dimensions can have clinical implications, over a relatively short time window of 10–15 years, indicating patients that potentially would require surgical intervention versus those in which the process of aortic dilation progresses at a slower rate. Similar values of aortic growth rate have been reported in the literature. AAo growth rate in BAV patients can range from 0.2 to 2.3 mm/year depending on patient characteristics.^24 25^ According to a transthoracic echocardiogram (TTE) study performed on an isolated BAV population of 133 adults, the mean growth rate was 0.3 mm/year at the sinuses and 0.6 mm/year at the level of the AAo, whereas RL valve fusion morphotype and AAo dilation phenotype where associated with slower aortic growth,^19^ in agreement with our results.

Our previous cross-sectional study focused exclusively on aortic morphology suggested that patients with reCoA tended to present increased aortic diameters compared with CoA patients with successful repair and no residual narrowing,^18^ yet current results did not reveal an association between reCoA and aortic growth rate. This may be partly due to a relatively short time window, nevertheless significant increases in aortic size were observed in other patients over this time. Aortic stenosis and regurgitation, as well as history of hypertension, were also not associated with aortic growth rate. It has been previously reported in the literature that BAV is a significant predictor of AAo complications in patients with CoA irrespectively of high blood pressure, and aortic dilation and dissection in CoA patients are not only explained by hypertension and valve dysfunction.^13^ Although BAV and CoA have been characterised as ‘two villainous cardiovascular lesions in cahoots’ with a similar pathophysiology that is part of a diffuse arteriopathy,^26^ yet the heterogeneity and abnormal haemodynamics characterising BAV disease make it difficult to identify the key mechanisms that should be investigated through more studies in BAV with and without CoA.^23 27^

This study focused exclusively on patients with BAV. Aortic growth in BAV patients is considerably more prevalent and faster compared with tricuspid aortic valve patients. Current, joint guidelines of the American College of Cardiology/American Heart Association and collaborating societies recommend elective repair for patients with/without BAV when the aortic diameter reaches ≥55 mm, or at 50 mm for those with BAV and: uncontrolled hypertension, family history of dissection and rapid growth rate >5 mm/year. The last indication includes presence of severe valve dysfunction (either stenosis/regurgitation) and an aortic diameter >45 mm.^28^ Therefore, given that growth rate drives surgical indication in BAV patients, refining the knowledge of its behaviour in association with CoA is warranted. Our study shows that the coexistence of these two entities does not necessarily imply a faster growth rate. As the matter of fact, the presence of CoA showed a slower growth rate despite associated risk factors such as history of hypertension.

Considering the indexed areas and the follow-up measurements from the current study, four patients in this population would be identified to be at risk of aortic dissection and requiring intervention, with area >10 cm^2^/m and 48–50 mm diameter.^21 29^ It should also be noted that our population is relatively young (average age 39 years overall) and while we cannot extrapolate information on growth rate beyond the time window that was studied, we cannot exclude further aortic dilation in these patients. This requires further analysis, not only by considering a wider timeframe, but potentially also making use of predictive modelling methodologies that may allow us to study aortic growth over time.

Finally, measurements were performed during both systole and diastole. While this may not reflect clinical practice, it beings to address the potentially dynamic nature of the problem, which should be addressed in the future in a separate study focusing on changes in aortic distensibility.

The study has the limitations of a retrospective design. Blood pressure data (actual cuff pressure at the time of CMR) and other risk factors (such as smoke and dyslipidaemia) were not available for all patients based on the available clinical data; however, history of hypertension was considered in the analysis. With regards to dimensional assessment, admittedly CMR imaging offers a limited spatial resolution compared with echocardiography and CT imaging, nevertheless our results agree with values of growth rate reported in the literature from echocardiographic studies.^19^ While TTE remains the technique of choice in the clinical follow-up of BAV patients, a comparison between TTE and CMR in measuring proximal AAo diameters in BAV patients showed that TTE measurements may be inaccurate in the presence of root asymmetry and RN fusion pattern.^30^ CMR imaging is increasingly utilised in clinical follow-up and assessment of these patients, particularly when aortic morphology cannot be accurately assessed by TTE. Also, in the case of aortic dilatation or aneurysmal enlargement, a CMR scan is recommended to fully evaluate the thoracic aorta, in terms of further assessing the severity of dilation and the involvement of the AAo.^17^

This study highlights the importance of studying aortic growth in BAV patients with and without associated CoA, since BAV-aortopathy is likely a reflection of BAV morphotype rather than CoA or its physiological effects. In this light, CoA patients with concomitant BAV disease should be considered as a separate group having two different disease phenotypes and receiving different monitoring and treatment from isolated CoA patients. In addition, this study confirms that patients with isolated BAV and RN fusion pattern may present substantially faster aortic root growth, whereas the more common aortic dilation phenotype and valve fusion pattern appeared as a more stable disease entity with slower progression. Therefore, patient stratification and identification of risk factors of aortic growth can lead to a more targeted prognosis and monitoring of the condition.
