# Supplementary material for: Determinants of aortic growth rate in patients with bicuspid aortic valve by cardiovascular magnetic resonance
Source: Open Heart. 2019 Nov 2;6(2):e001095. doi: 10.1136/openhrt-2019-001095 (PMC6861085; doi:10.1136/openhrt-2019-001095)
Supplement: Supplementary data [file openhrt-2019-001095supp001.pdf]

Supplementary Material

Table A: Diameters, area values and aortic growth rate as indexed area of AAo and SoV both in end-systole and end-diastole, corresponding to the subgroups of CoA, NoCoA, reCoA, vale type 0, type 1 with RL cusp fusion and RN cusp fusion.

**Variables**	**SoV ES**					**SoV ED**				
	**Diameter time 1 (mm, mean ± SD)**	**Diameter time 2 (mm, mean ± SD)**	**Area time 1 (cm2, mean ± SD)**	**Area time 2 (cm2, mean ± SD)**	**Growth rate area (area, cm^2/m*yr, mean ± SD)**	**Diameter time 1 (mm, mean ± SD)**	**Diameter time 2 (mm, mean ± SD)**	**Area time 1 (cm2, mean ± SD)**	**Area time 2 (cm2, mean ± SD)**	**Growth rate area (area, cm^2/m*yr, mean ± SD)**
**CoA**	32.1±5.1	32.8±5.4	826.4±257.6	863.4±285.3	0.05±0.09	31.2±5.3	31.9±5.5	785.7±261.2	821.8±282.7	0.05±0.09
**NoCoA**	35.6±4.5	36.5±4.6	1004.4±255.2	1061.5±266.8	0.2±0.3	34.7±4.6	35.6±4.7	959.9±252.7	1010.9±265.7	0.1±0.2
**ReCoA**	31.6±5.2	32.2±5.5	806.0±272.9	838.6±300.9	0.05±0.09	30.8±5.4	31.4±5.6	765.7±274.5	796.3±294.1	0.04±0.06
**Valve type 0**	32.4±5.3	33.1±5.6	839.4±278.8	880.7±308.3	0.08±0.1	31.3±5.6	32.1±5.8	786.5±286.0	828.0±313.9	0.08±0.2
**Valve type 1, RL fusion**	33.9±5.2	34.7±5.4	919.3±396.6	919.3±277.2	0.1±0.2	33.1±5.3	33.8±5.5	878.1±276.1	920.9±294.3	0.08±0.2
**Valve type 1, RN fusion**	35.6±4.1	36.9±3.9	1005.6±233.7	1079.5±229.4	0.3±0.5	34.9±4.2	36.1±4.1	966.6±232.3	1031.1±236.2	0.2±0.3

Variables	AAo ES					AAo ED				
	Diameter time 1 (mm, mean ± SD)	Diameter time 2 (mm, mean ± SD)	Area time 1 (cm2, mean ± SD)	Area time 2 (cm2, mean ± SD)	Growth rate area (area, $cm^2/m*yr$, mean ± SD)	Diameter time 1 (mm, mean ± SD)	Diameter time 2 (mm, mean ± SD)	Area time 1 (cm2, mean ± SD)	Area time 2 (cm2, mean ± SD)	Growth rate area (area, $cm^2/m*yr$, mean ± SD)
**CoA**	30.1±6.8	31.3±7.1	746.5±328.3	805.9±356.9	0.08±0.1	28.7±7.0	29.8±7.2	681.1±321.5	737.2±344.6	0.08±0.1
**NoCoA**	37.6±5.5	38.8±5.2	1134.3±316.1	1200.1±313.6	0.19±0.5	36.4±5.6	37.5±5.3	1060.8±317.6	1124.1±311.1	0.2±0.4
**ReCoA**	29.1±6.18	30.3±6.7	694.7±289.7	752.5±329.8	0.08±0.1	27.7±6.3	29.0±6.8	633.4±281.3	692.9±320.7	0.08±0.1
**Valve type 0**	32.4±6.2	33.4±6.4	853.2±298.3	905.1±321.3	0.1±0.1	31.1±6.3	32.1±6.6	788.1±289.6	840.5±318.5	0.1±0.1
**Valve type 1, RL fusion**	34.0±7.4	35.1±7.5	948.5±388.3	1011.6±403.6	0.1±0.2	32.6±7.6	33.8±7.6	880.4±386.0	940.4±396.6	0.1±0.2
**Valve type 1, RN fusion**	38.5±4.3	39.7±3.9	1177.3±264.8	1245.8±247.6	0.4±1.1	37.3±4.4	38.4±3.9	1104.2±266.2	1169.1±241.5	0.3±0.7

CoA= coarctation; RL= right coronary and left coronary; RN= right coronary and non-coronary.
